# Urinary alpha-1 antitrypsin and CD59 glycoprotein predict albuminuria development in hypertensive patients under chronic renin-angiotensin system suppression

**DOI:** 10.1186/s12933-016-0331-7

**Published:** 2016-01-16

**Authors:** Laura Gonzalez-Calero, Marta Martin-Lorenzo, Fernando de la Cuesta, Aroa S. Maroto, Montserrat Baldan-Martin, Gema Ruiz-Hurtado, Helena Pulido-Olmo, Julian Segura, Maria G. Barderas, Luis M. Ruilope, Fernando Vivanco, Gloria Alvarez-Llamas

**Affiliations:** Departamento de Inmunologia, Laboratorio de Inmunoalergia y Proteomica, IIS-Fundacion Jimenez Diaz, UAM, REDinREN, Avda Reyes Catolicos 2, 28040 Madrid, Spain; Laboratorio de Fisiopatologia Vascular, Hospital Nacional de Paraplejicos SESCAM, Toledo, Spain; Unidad de Hipertension, Instituto de Investigacion i + 12, Hospital Universitario 12 de Octubre, Avenida de Córdoba s/n, 28041 Madrid, Spain; Instituto Pluridisciplinar, Universidad Complutense de Madrid, Madrid, Spain; Departamento de Bioquimica y Biologia Molecular I, Universidad Complutense de Madrid, Madrid, Spain

**Keywords:** Albuminuria, Alpha-1 antitrypsin, Cardiovascular risk, CD59, Hypertension, Urine, Markers

## Abstract

**Background:**

Hypertension is a multi-factorial disease of increasing prevalence and a major risk factor for cardiovascular mortality even in the presence of adequate treatment. Progression of cardiovascular disease (CVD) occurs frequently during chronic renin-angiotensin-system (RAS) suppression, and albuminuria is a marker of CV risk. High prevalence of albuminuria in treated hypertensive patients has been demonstrated, but there are no available markers able to predict evolution. The aim of this study was the identification of novel indicators of albuminuria progression measurable in urine of diabetic and non-diabetic patients.

**Methods:**

1143 hypertensive patients under chronic treatment were followed for a minimum period of 3 years. Among them, 105 diabetic and non-diabetic patients were selected and classified in three groups according to albuminuria development during follow-up: (a) patients with persistent normoalbuminuria; (b) patients developing de novo albuminuria; (c) patients with maintained albuminuria. Differential urine analysis was performed by 2D gel electrophoresis (2D-DIGE) and further confirmed by liquid chromatography-mass spectrometry. Non-parametric statistical tests were applied.

**Results:**

CD59 glycoprotein and alpha-1 antitrypsin (AAT) resulted already altered in patients developing albuminuria de novo, with a similar response in those with maintained albuminuria. A prospective study in a sub-group of normoalbuminuric patients who were clinically followed up for at least 1 year from urine sampling, revealed CD59 and AAT proteins significantly varied in the urine collected from normoalbuminurics who will negatively progress, serving as predictors of future albuminuria development.

**Conclusions:**

CD59 and AAT proteins are significantly altered in hypertensive patients developing albuminuria. Interestingly, CD59 and AAT are able to predict, in normoalbuminuric individuals, who will develop albuminuria in the future, being potential predictors of vascular damage and CV risk. These findings contribute to early identify patients at risk of developing albuminuria even when this classical predictor is still in the normal range, constituting a novel strategy towards a prompt and more efficient therapeutic intervention with better outcome.

**Electronic supplementary material:**

The online version of this article (doi:10.1186/s12933-016-0331-7) contains supplementary material, which is available to authorized users.

## Background

Cardiovascular disease continues being the first cause of death even in the presence of adequate treatment, where renin-angiotensin-system (RAS) blockade is fundamental and has to be chronically maintained. Albuminuria is a marker of cardiovascular damage, and maintenance of albuminuria or development of de novo albuminuria during adequate treatment is a marker of cardiovascular disease progression and potentially of renal function worsening [1–4]. In fact, worse clinical outcomes have been observed in individuals with high albuminuria and preserved estimated glomerular filtration rate (eGFR) than for those showing reduced eGFR and no albuminuria [5].

The ability of renin-angiotensin system (RAS) suppression to reduce albuminuria has been amply demonstrated. It is well established that RAS suppression is required in patients with increased amounts of albumin in urine with the double objective of facilitating blood pressure (BP) control while diminishing the amount of albumin in urine beyond the limit obtained by BP drop [6, 7]. However, during chronic suppression of RAS progression of cardiovascular damage is very frequent, and here again the presence of albuminuria is an indicator of bad prognosis. Our group and others have shown the development of albuminuria in patients receiving chronic suppression of the RAS [3, 8]. In our experience development of new-onset high albuminuria has been observed in 16.1 % of normoalbuminuric patients during a follow-up of 3 years [3]. The development of new-onset albuminuria in our patients under chronic RAS suppression is accompanied by a characteristic pattern of increased oxidative stress [9]. The need to discover predictors of the development and the ulterior worsening of CV and renal prognosis is required [10, 11].

Omics-based approaches are powerful strategies to investigate novel markers of disease aimed to improve current diagnosis, predict patient prognosis or define novel therapeutic targets, which have been successfully applied in kidney pathology [12–15]. Urine has been investigated, in particular, to study renal physiology and kidney diseases, as it represents a combination of both plasma ultrafiltrate and urinary tract proteins, including glomerular filtered plasma proteins and soluble proteins secreted by epithelial cells and microvesicles as exosomes. Complementary to plasma or serum, urine is an ideal non-invasive biofluid, quite stable and subject to minimal degradation in the bladder and urinary tract, and a rich source of potential markers of disease. Urine composition not only reflects normal kidney function but also contains specific kidney produced proteins which may be altered in response to underlying physiopathology [16]. Our group and others have investigated urine in the search for novel molecular targets of potential use in the clinical setting from a diagnosis or prognosis point of view in the context of cardiovascular disease [17]. Albuminuria is also a predictor of renal damage and urinary omics has also been applied to diabetic nephropathy [18], acute kidney injury [19], and chronic kidney disease [20] among others [21]. Here, we applied first differential proteomic analysis to investigate early urinary changes in albuminuria progression in non-diabetic hypertensive patients with chronically RAS suppression. A highly sensitive, specific and high-throughput methodology was additionally implemented to confirm and quantify most dramatic molecular changes taking place in urine and here identified, as predictor markers of albuminuria progression, both in diabetics and non-diabetics.

## Methods

### Patient recruitment

Patient selection and classification was previously described in an initial paper showing the development of high albuminuria in patients during chronic RAS suppression [3]. Briefly, 1143 patients were followed for a minimum period of 3 years with visits to the Hypertension Unit, Hospital Universitario 12 de Octubre, Madrid, at least, every 6 months. After that, the patients continued with their annual revisions. One hundred and five hypertensive patients (66 non diabetic and 39 diabetic) with or without albuminuria were recruited between January 2012 and June 2013. Patients were classified according to high albuminuria development during follow-up in 3 groups: (a) patients with persistent normoalbuminuria (N); (b) patients developing de novo albuminuria during follow-up (dnHA); (c) patients with maintained albuminuria during follow-up (MHA). A control group of urine samples from healthy individuals (C) was also included to evaluate potential differences attributed to hypertension itself. Details about specific cohorts included in the discovery or confirmation phase are given in the following sections. Patients with renal diseases as potential cause of hypertension were excluded. The study was conducted according to recommendations of Declaration of Helsinki and approved by the Ethics Committee of the Hospital 12 de Octubre. Informed consent was requested from subjects prior to inclusion in the study.

### Differential proteomics analysis (discovery phase)

Individual urine samples from a total of 15 patients and six healthy subjects were used in the discovery phase [5–6 individual samples were pooled per group (C, N, dnHA and MHA)]. All subjects were non-diabetic to avoid diabetes as a background pathology in this first discovery phase. Table 1 compiles baseline characteristics of patients included in this phase, showing perfectly matched groups with no significant differences in any of the variables among patient groups (Additional file 1: Table S1 shows additional information of baseline patients’ medication). Differential proteomics analysis in urine was performed by 2D-DIGE (GE Healthcare) as previously published [22–24]. Gels were scanned using a Typhoon 9400 Variable Mode Imager (GE Healthcare) and spot maps (gel images) were processed, analyzed and compared using the DeCyder Differential Analysis Software version 6.5 (GE Healthcare). An ANOVA test was performed with the expression data of each spot. Differential proteins were tryptic digested and identified in a MALDI-TOF/TOF mass spectrometer 4800 plus Proteomics Analyzer (Applied Biosystems. MDS Sciex, Toronto, Canada) with 4000 Series Explorer™v 3.5 Software (ABSciex) as previously described [25] with a probability score greater than the one fixed by Mascot as being significant (*p* value <0.05).

**Table 1:** Baseline patients' characteristics used in the discovery phase

	N (n = 5)	dnHA (n = 5)	MHA (n = 5)	P value
Age (years)	58 ± 7	58 ± 7	62 ± 5	0.463
Sex (male), %	60	60	60	>0.999
BMI (kg/m^2^)	30 ± 3	29 ± 1	28 ± 4	0.650
Current smoking, %	0	40	20	0.725
Total cholesterol (mg/dl)	188 ± 32	157 ± 15	175 ± 18	0.159
Triglycerides (mg/dl)	114 ± 43	112 ± 68	90 ± 27	0.765
HDL cholesterol (mg/dl)	49 ± 12	51 ± 23	58 ± 12	0.497
LDL cholesterol (mg/dl)	116 ± 34	83 ± 13	99 ± 12	0.150
Glycaemic (mg/dl)	91 ± 11	101 ± 7	92 ± 8	0.190
Uric acid (mg/dl)	5.6 ± 1.7	6.1 ± 1.8	4.6 ± 1.2	0.330
Creatinine clearance rate (mg/ml)	87 ± 24	112 ± 61	89 ± 28	0.876
eGFR (ml/min/1.73 m^2^)	83 ± 10	87 ± 28	88 ± 17	0.536
Systolic blood pressure (mmHg)	132 ± 16	138 ± 8	126 ± 15	0.390
Diastolic blood pressure (mmHg)	84 ± 14	83 ± 10	81 ± 7	0.869
ACR (mg/g)	3.8 ± 1.7	74 ± 33	75 ± 90	0.001
Diabetes mellitus, %	0	0	0	>0.999

Values expressed as mean ± SD or percentages (%)

*BMI* body mass index, *HDL* high-density lipoprotein cholesterol, *LDL* low-density lipoprotein cholesterol, *N* normoalbuminuria, *dnHA* de novo high albuminuria, *MHA* maintained high albuminuria

### Mass spectrometry-based analysis in a confirmation cohort (validation phase)

As previously published by our group and others [17, 18, 26] we used the SRM-LC-MS/MS methodology to confirm differential proteins identified in the discovery phase. For such purpose, we collected urine samples from a different individuals’ cohort to that used in the discovery phase composed by 90 patients (39 diabetic and 51 non-diabetic) and 18 healthy subjects. Table 2 compiles the baseline characteristics of this validation cohort (Additional file 1: Table S2 shows additional information of baseline patients’ medication). The three groups are comparable with marginal differences for total cholesterol, HDL cholesterol and uric acid. In brief, urinary proteins were tryptic digested and analyzed in a 6460 triple quadrupole mass spectrometer on-line connected to nano-chromatography (1200 Series, Agilent Technologies) in a Chip-format configuration (ChipCube interface, ProtID Zorbax 300B-C18-5 µm chip, 43 × 0.075-mm analytical column and 40 nL enrichment column, Agilent Technologies). The system was controlled by Mass Hunter Software (v4.0 Agilent Technologies). Theoretical SRM transitions were designed using Skyline (v.1.1.0.2905) and peptide specificity was confirmed by protein blast. Samples were analyzed in duplicate.

**Table 2:** Baseline patients' characteristics used as confirmation cohort

	N (n = 47)	dnHA (n = 20)	MHA (n = 23)	P value
Age (years)	65 ± 11	69 ± 7	65 ± 12	0.382
Sex (male), %	34	70	61	0.012
BMI (kg/m^2^)	31 ± 5	30 ± 4	31 ± 5	0.796
Current smoking, %	13	15	13	0.969
Total cholesterol (mg/dl)	186 ± 29	166 ± 27	170 ± 31	0.035
Triglycerides (mg/dl)	119 ± 53	130 ± 70	139 ± 74	0.488
HDL cholesterol (mg/dl)	56 ± 13	51 ± 9	44 ± 12	0.0003
LDL cholesterol (mg/dl)	106 ± 28	90 ± 19	100 ± 24	0.088
Glycaemic (mg/dl)	118 ± 42	123 ± 26	119 ± 34	0.387
Uric acid (mg/dl)	4.9 ± 1.5	6.3 ± 1.5	6.9 ± 1.7	<0.0001
Creatinine clearance rate (mg/ml)	101 ± 40	97 ± 47	76 ± 41	0.138
eGFR (ml/min/1.73 m^2^)	81 ± 18	68 ± 19	64 ± 29	0.024
Systolic blood pressure (mmHg)	138 ± 18	139 ± 22	140 ± 28	0.975
Diastolic blood pressure (mmHg)	81 ± 11	81 ± 11	82 ± 17	0.993
ACR (mg/g)	11 ± 13	211 ± 388	662 ± 910	<0.0001
Diabetes mellitus, %	32	60	52	0.066

Values expressed as mean ± SD or percentages (%)

*BMI* body mass index, *HDL* high-density lipoprotein cholesterol, *LDL* low-density lipoprotein cholesterol, *N* normoalbuminuria, *dnHA* de novo high albuminuria, *MHA* maintained high albuminuria

### Statistical methods

Nonparametric Kruskal–Wallis test with Dunn’s multiple comparisons post-test or nonparametric Mann–Whitney, when only two groups are compared (progressors and non-progressors), were applied by means of GraphPad Prism 6 (version 6.01) software to calculate statistically significant differences of the values between different groups. We applied the ROUT method to detect outliers based on the false discovery rate (FDR), setting Q value to 5 %. Receiver operating characteristic (ROC) curves were generated using GraphPad Prism 6 software (confidence level 95 %).

## Results

**Fig. 1:**
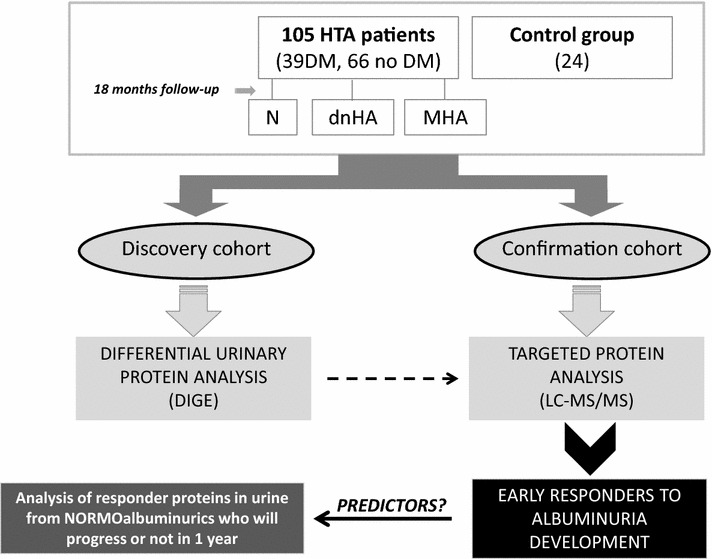
Schematic view of workflow. HTA hypertensive; DM diabetes mellitus; N normoalbuminuric; dnHA de novo high albuminuria; MHA mantained high albuminuria; DIGE differential gel electrophoresis; LC-MS/MS liquid chromatography mass spectrometry in tandem

In Fig. 1, a schematic workflow summarizes the whole study. We first investigate most significant molecular changes in urine in response to different albuminuria progression affecting hypertensive patients chronically RAS suppressed according to groups division detailed in methods section: C, N, dnHA, MHA (Tables 1 , 2, Additional file 1: Tables S1 and S2).

One-way ANOVA revealed six proteins significantly altered: α-1-B glycoprotein, α-1 antitrypsin (AAT or SERPINA1), tetranectin (TNA or CLEC3B), CD59 glycoprotein (CD59), protein AMBP and zinc-α-2-glycoprotein. Table 3 shows trends observed for inter-groups comparisons, where arrows reflect increase or diminish in the group located in the upper part of the ratio. Further details can be found in Additional file 1: Table S3 and a representative image of 2D-DIGE gel is shown in Additional file 1: Figure S1. These initial data composed a training set of molecular changes taking place in urine with different responses to hypertension and albuminuria progression. Principal component analysis (PCA) showed perfect grouping of individual urines according to healthy condition (C), hypertension without albuminuria (N), dnHA or MHA. Interestingly, individuals with progressing albuminuria (dnHA) are those most separated from the normoalbuminuria status, as corresponds to such unstable situation (Additional file 1: Figure S2).

**Table 3:** Proteins significantly altered (DIGE, discovery phase) in response to different albuminuria development or progression

Protein name	Gene name	Accession number (UniProt)	N/C	dnHA/C	MHA/C	dnHA/N	MHA/N	MHA/dnHA	1-ANOVA
α-1-beta glycoprotein	A1BG	P04217	↑	↑	↑	↑	↑	↓	0.00042
α-1-antitrypsin	AAT	P01009	↓	↑	↑	↑	↑	↓	0.018
Tetranectin	TNA	P05452	↑	↓	↓	↓	↓	↑	0.033
AMBP protein	AMBP	P02760	↓	↓	↓	↓	↑	↑	0.012
CD59 glycoprotein	CD59	P13987	↑	↓	↓	↓	↓	↑	0.0022
Zinc-α2 glycoprotein	AZGP1	P25311	↑	↑	↑	↑	↑	↓	0.016

Arrows reflect increase or diminish in the group located in the upper part of the ratio

**Fig. 2:**
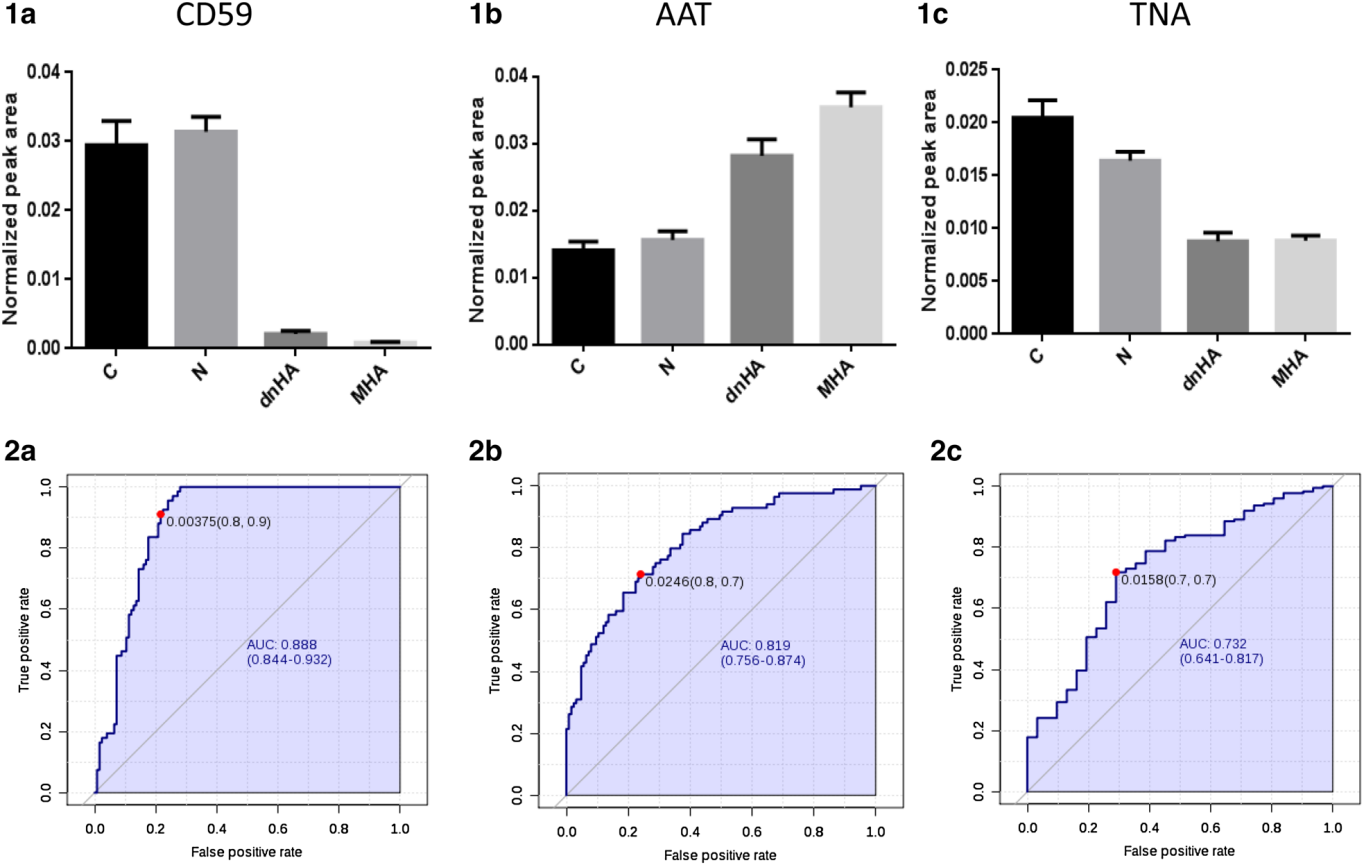
CD59 (**1a**, **2a**), AAT (**1b**, **2b**) and TNA (**1c**, **2c**) urinary response in healthy and hypertensive patients chronically RAS suppressed at different stages of albuminuria progression. *Graphs* show normalized peak area from SRM-LC-MS/MS data. *ROC curves* evaluate response to albuminuria condition (CD59 and AAT) or hypertension (TNA)

An adequate validation of the previous findings was accomplished by a mass spectrometry-based approach in the urine from a new cohort of 108 individuals, to confirm previous data and define a molecular fingerprint most strongly associated to hypertension and albuminuria progression. Molecular alterations previously found were confirmed for CD59, AAT and TNA (Fig. 2). CD59 glycoprotein and AAT showed an altered response already in those individuals who developed dnHA (CD59 decreases and AAT increases) who, according to these two proteins, behave as patients with MHA. Differently, TNA shows a decreasing trend in response to hypertension condition. ROC curves are shown for the three proteins (Fig. 2). CD59 and AAT show good sensitivity and selectivity (AUC = 0.888 and 0.819, respectively) in classifying normoalbuminuric from high albuminuric (de novo or maintained). Tetranectin allows discriminating between control and hypertensive subjects (AUC = 0.732).

**Fig. 3:**
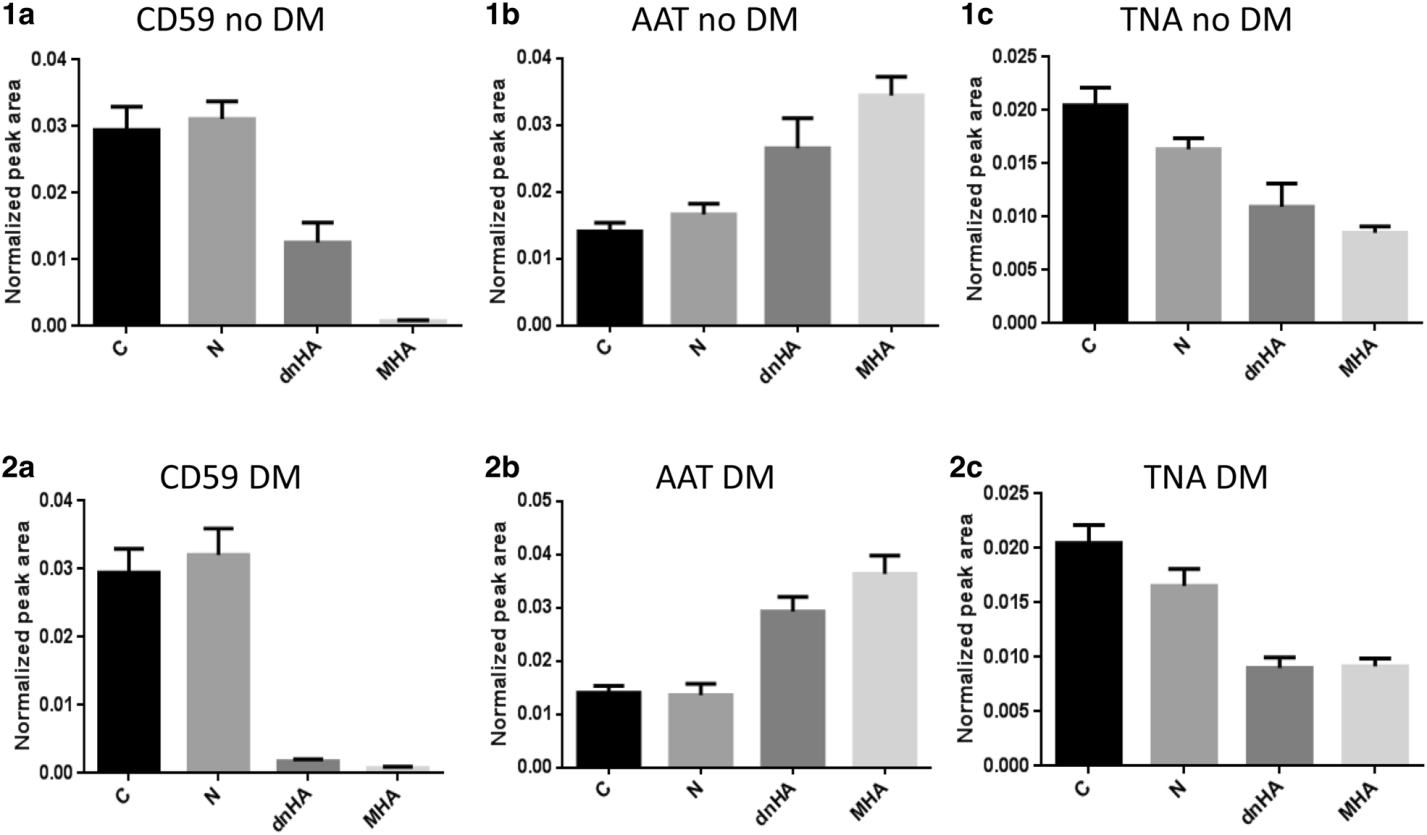
Effect of diabetes mellitus on urinary CD59 (**1a**, **2a**), AAT (**1b**, **2b**) and TNA (**1c**, **2c**). dnHA diabetic patients show CD59 levels closer to those observed for individuals with MHA

It is worthwhile to note that discovery of novel potential markers associated to albuminuria progression in chronically RAS suppressed patients was approached including, exclusively, non-diabetic patients. The number of recruited individuals was highly increased in the validation phase, including 36 % of diabetic subjects. However, no significant differences were found attributed to the diabetic condition as can be seen in Fig. 3, apart from a slightly more pronounced behavior towards MHA observed for CD59 in diabetic dnHA.

**Fig. 4:**
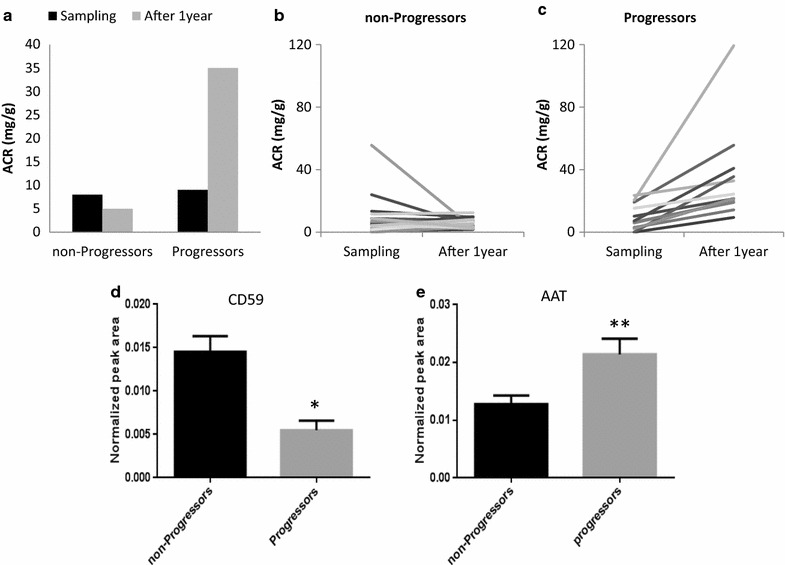
CD59 and AAT predict progression of albuminuria in normoalbuminuric patients. Albuminuria was evaluated at sampling and at least 1 year later and individuals were classified as non-progressors or progressors (**a**, **b**, **c**). Levels of CD59 and AAT in urine when those individuals were normoalbuminuric can already distinguish both groups, anticipating future development or not of albuminuria (**d**, **e**)

To further evaluate these proteins as predictors of high albuminuria development, we performed a prospective study. A sub-group of those hypertensive patients, who were classified as normoalbuminuric during urine sampling for the study, were clinically followed up for at least 1 year from urine sampling (when all them showed normal albuminuria values). From 37 monitored patients 25 remained as normoalbuminuric, meanwhile 12 progressed to dnHA (observed increase in at least 10 units of ACR). In Fig. 4a–c, albuminuria evolution for stable and progressed individuals can be seen, showing similar values at the time of the study for all them, independently of their individual progression. When CD59 and AAT protein levels were measured in urine collected when no differences were observed in albuminuria, significant variation for both proteins were already detected, serving as predictors of future albuminuria evolution (Fig. 4d, e).

## Discussion

High albuminuria is a known predictor of increased cardiovascular risk and future cardiovascular events [4, 27]. As such, albuminuria has to be considered more as a marker of damage than as a risk factor [28]. Recent studies in hypertensive individuals with low-moderate CV risk showed that the presence of increased albumin excretion at baseline or de novo development of albuminuria during follow-up is linked to a higher CV risk independently of other CV risk factors [3, 8]. Renin-angiotensin system blockade is the most effective therapeutic approach to treat albuminuria with a double aim of controlling BP and preventing or diminishing albuminuria. However, regression of albumin excretion in response to treatment does not necessarily imply improved CV prognosis [29–31]. In fact the decrease in albuminuria needs to be superior to 50 % in order to see an improvement in prognosis [32]. Hypertension is associated with end-organ damage, particularly affecting heart, vessels and kidneys, and evidence of organ damage implies significant worsening of CV prognosis even in the presence of an apparently moderate global CV risk calculated according to SCORE [33]. Albuminuria is partially a consequence of high blood pressure but also other factors such as systemic inflammation and endothelial dysfunction participate in its appearance as well as on the progressive decay in glomerular filtration rate [34]. Amelioration of the damage leading to the development of albuminuria and other forms of target organ damage does not necessarily translate into a reduction in CV risk, even so assessment of end-organ damage is totally required in hypertensives because it allows a more adequate adjustment of individual therapeutic intervention [29].

In previous studies from our group, we showed new onset of albuminuria in RAS suppressed hypertensives in 16.1 % of cases. Alterations at protein level in urine in response to hypertension and salt sensitivity have been reported [35]. In this work we aimed the identification of molecular indicators in urine linked to albuminuria development, particularly in non-diabetic hypertensives under chronic RAS suppression. Pursuing an early management aimed to prevent negative progression and irreversible damage, identified responder proteins were also investigated in urine from normoalbuminuric patients. We identified a urine molecular panel of proteins responding differently to albuminuria development in our patients: CD59, AAT and TNA. CD59 and AAT already showed altered levels in urine in patients developing high albuminuria during the follow-up before the rise in the urinary protein took place, thus serving as predictors of the progression to high albuminuria. CD59 was found to be down-regulated in urine before and after the rise in albumin excretion in urine. CD59 inhibits the membrane attack complex (MAC) to protect cells in an inflammatory scenario. CD59 and decay accelerating factor (DAF) are synthesized by mesangial and epithelial cells, and complement activation translates into higher levels of both in a number of renal diseases, among them those with glomerular injury [36, 37]. However this increment did not occur in other renal diseases particularly those with tubulo-interstitial damage and a similar situation could happen in our patients [36]. Interestingly, a slight increase in blood pressure was observed in CD59 knockout mice [38]. In agreement, a reduced expression of CD59 in endothelial cells from hypertensive patients has been described, suggesting a potential role in development of hypertension and increased CV risk [39]. Similarly a diminished urinary CD59 has been found in diabetics (normo and microalbuminuric) potentially contributing to the increased CV risk in these patients [15]. In our study, CD59 showed low urinary levels in patients developing de novo albuminuria. The decrease was more pronounced in diabetics. Additionally, urinary CD59 was shown to differentiate those hypertensive patients with normal albuminuria who will negatively progress. Thus, these observations suggest that decreased urinary levels of CD59 can be considered as an early indicator of vascular damage and progression of atherosclerosis in chronically RAS suppressed hypertensives [40, 41].

AAT is an inflammation-sensitive plasma protein (ISP) found increased in urine of individuals with diabetic nephropathy [42, 43], in diabetics with normo, micro or macroalbuminuria [44, 45] and in serum of obese individuals with metabolic syndrome [46], pointing to a role for this protein in diabetic complications. AAT is also increased in urine from patients with essential and secondary hypertension, but without a correlation with albumin excretion in urine, which supports the idea of glomerular filtration and AAT local production in damaged kidney as confluencing mechanisms responsible for increased levels in urine [47]. Our study goes a step further, non-diabetics show increased urinary AAT levels in patients developing de novo albuminuria as well as in those with maintained albuminuria. More importantly, increased level in urine from normoalbuminurics enables to predict a negative progression. Thus, if albuminuria is considered an indicator of (renal) end-organ damage, we hypothesize that the increase in AAT observed in normoalbuminuric hypertensives with negative prognosis (albuminuria development) can be considered as an earlier evidence, supporting the idea of local synthesis in response to inflammation.

The existence of a cross-talk between cardiovascular and renal systems, accompanied by activation of the coagulation system, increased inflammation and altered autoimmune reactivity has been shown [48–51]. In particular, AAT inhibits activated protein C and plasminogen activator (PA) which promotes the conversion of plasminogen to plasmin which in turn acts to degrade fibrin. Tetranectin also enhances plasminogen activation and plasma TNA levels have been inversely associated with coronary artery disease [52]. Increased levels in AAT and decreased levels in TNA in response to albuminuria point in the same direction, taking in an impairment of the regulation of the anticoagulation system and in agreement with a potential therapeutical intervention over plasminogen activator inhibitor (PAI-1) in hypertensives [53].

**Fig. 5:**
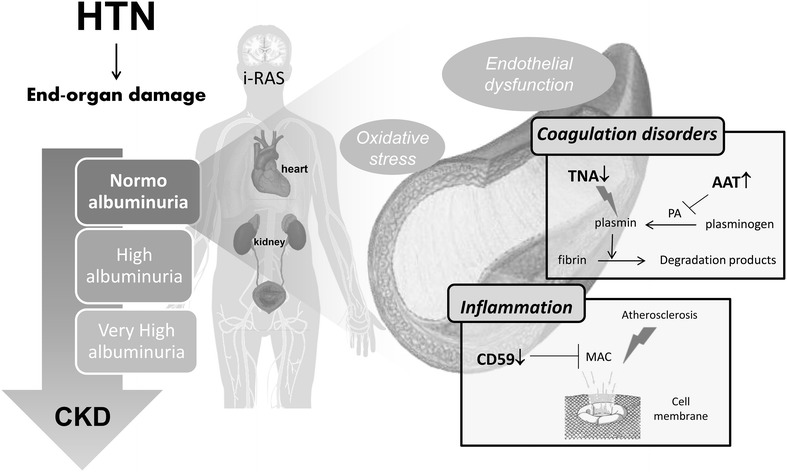
Main alterations in response to albuminuria progression point to inflammation and coagulation disorders as main biological pathways involved. Differences observed already in normoalbuminuria condition reveal subjacent mechanisms associated with cardiovascular risk taking place already in asymptomatic stages

These altered responses point in one direction, linking vascular dysfunction and kidney disease (Fig. 5) [54]. Molecular alterations found here not only respond to high albuminuria clinical condition, but interestingly, they are already altered in normoalbuminuric patients chronically treated who will further progress to high albuminuria. Thus, molecular disorders related to vascular dysfunction, atherosclerosis progression and cardiovascular risk are already taking place at earlier stages when albuminuria is still in the normal range.

This study accomplishes the requirements for a proteomics study in urine with a small initial cohort of non-diabetic patients for the discovery phase. The group used in the validation cohort was significantly more numerous and including both diabetic and non-diabetic individuals. However, as we have demonstrated here, the presence of diabetes did not significantly influence our findings.

## Conclusions

Our data were obtained in hypertensive patients on chronic RAS suppression. CD59 and AAT proteins are significantly altered in hypertensive patients (diabetics and non-diabetics) developing albuminuria de novo, despite being treated. Additionally, CD59 and AAT are able to predict in normoalbuminuric individuals who will develop albuminuria in the future. These findings contribute to early identification of patients at risk of developing albuminuria even when this classical predictor is still in the normal range, that is patients in whom cardiovascular and renal disease progresses despite RAS suppression. Our data set the basis for a novel strategy towards a prompt and more efficient therapeutic intervention with better outcome, i.e. identifying patients at risk of developing albuminuria and in whom enhancement of RAS blockade, probably by using a mineralocorticoid receptor antagonist, may be indicated.

## Additional file

10.1186/s12933-016-0331-7 Representative image of 2D-DIGE gel. **Figure S2**. Principal component analysis (PCA) graph. Each dot represents a urine sample used in the discovery phase (DIGE analysis). **Table S1.** Baseline medication for those patients including in the discovery phase. Data are expressed as percentages (%). ACEi: angiotensin converting enzyme inhibitors; ARB: angiotensin receptor blockers. N: normoalbuminuria; dnHA: de novo high albuminuria; MHA: maintained high albuminuria. **Table S2.** Baseline medication for those patients including in the validation phase. Data are expressed as percentages (%). ACEi: angiotensin converting enzyme inhibitors; ARB: angiotensin receptor blockers. N: normoalbuminuria; dnHA: de novo high albuminuria; MHA: maintained high albuminuria. **Table S3.** Proteins identifiedper gel spot with significant alteration (one-way ANOVA). The table shows the number of unique peptides identified, % sequence coverage and trends observed for each protein between compared groups (increase or decrease in the group located in the upper part of the ratio). Two spots contain a mixture of two proteins each, thus changes in expression forthose spots cannot be attributed to any of the two proteins initially. When one protein was identified in several spots, observed variations between groups followed the same trend (e.g. CD59 and alpha-1-antitrypsin). **Table S4.** SRM-LCMS/MS analysis conditions for those proteins confirmed in the validation phase with statistical signification (ANOVA <0.0001). Details of protein transitions (precursor and fragments masses), collision energy and peptide sequences are included.
