# Supplementary material for: Urinary alpha-1 antitrypsin and CD59 glycoprotein predict albuminuria development in hypertensive patients under chronic renin-angiotensin system suppression
Source: Cardiovasc Diabetol. 2016 Jan 16;15:8. doi: 10.1186/s12933-016-0331-7 (PMC4715311; doi:10.1186/s12933-016-0331-7)

**Supplemental data**

**Table S1.** Baseline medication for those patients including in the discovery phase. Data are expressed as percentages (%). ACEi: angiotensin converting enzyme inhibitors; ARB: angiotensin receptor blockers. N: normoalbuminuria; dnHA: *de novo* high albuminuria; MHA: maintained high albuminuria.

|  | **N (n=5)** | **dnHA (n=5)** | **MHA (n=5)** | **P-value** |
| --- | --- | --- | --- | --- |
| **Antihypertensives, %** |  |  |  |  |
| **ACEi** | 0 | 0 | 40 | 0.286 |
| **ARB** | 100 | 100 | 60 | 0.286 |
| **Diuretic** | 100 | 80 | 60 | 0.725 |
| **Calcium channel blocker** | 40 | 20 | 40 | >0.999 |
| **Beta blocking agent** | 40 | 0 | 40 | 0.450 |
| **Alpha blocking agent** | 0 | 20 | 0 | >0.999 |
| **Other treatments, %** |  |  |  |  |
| **Anticoagulant agent** | 0 | 20 | 20 | >0.999 |
| **Lipid lowering agents** | 60 | 60 | 80 | >0.999 |
| **Antidiabetic agent** | 0 | 0 | 0 | >0.999 |
| **Antialdosteronics** | 0 | 20 | 0 | >0.999 |
| **Hypouricemics** | 0 | 20 | 0 | >0.999 |

**Table S2.** Baseline medication for those patients including in the validation phase. Data are expressed as percentages (%). ACEi: angiotensin converting enzyme inhibitors; ARB: angiotensin receptor blockers. N: normoalbuminuria; dnHA: *de novo* high albuminuria; MHA: maintained high albuminuria.

|  | **N (n=47)** | **dnHA (n=20)** | **MHA (n=23)** | **P-value** |
| --- | --- | --- | --- | --- |
| **Antihypertensives, %** |  |  |  |  |
| **ACEi** | 17 | 15 | 13 | 0.909 |
| **ARB** | 77 | 70 | 74 | 0.851 |
| **Diuretic** | 49 | 45 | 56 | 0.738 |
| **Calcium channel blocker** | 62 | 55 | 74 | 0.418 |
| **Beta blocking agent** | 26 | 30 | 26 | 0.929 |
| **Alpha blocking agent** | 15 | 35 | 26 | 0.173 |
| **Other treatments, %** |  |  |  |  |
| **Anticoagulant agent** | 38 | 40 | 30 | 0.768 |
| **Lipid lowering agents** | 79 | 65 | 78 | 0.464 |
| **Antidiabetic agent** | 26 | 40 | 39 | 0.368 |
| **Antialdosteronics** | 19 | 10 | 17 | 0.654 |
| **Hypouricemics** | 6 | 20 | 22 | 0.129 |

**Table S3.** Proteins identified per gel spot with significant alteration (one-way ANOVA). The table shows the number of unique peptides identified, % sequence coverage and trends observed for each protein between compared groups (increase or decrease in the group located in the upper part of the ratio). Two spots contain a mixture of two proteins each, thus changes in expression for those spots cannot be attributed to any of the two proteins initially. When one protein was identified in several spots, observed variations between groups followed the same trend (e.g. CD59 and alpha-1-antitrypsin).

| **Spots** | **Accesion number (UniProt)** | **Protein name** | **Gene name** | **N/C** | **dnHA/C** | **MHA/C** | **dnHA/N** | **MHA/N** | **MHA/dnHA** | **ANOVA** | **Unique peptides** | | **Sequence Coverage (%)** |
| --- | --- | --- | --- | --- | --- | --- | --- | --- | --- | --- | --- | --- | --- |
| 1 | **P04217** | alpha1-beta glycoprotein | **A1BG** | ↑ | ↑ | ↑ | ↑ | ↑ | ↓ | 0.00042 | 12 | 36 | |
| 2 | **P01009** | alpha1-antitrypsin | **AAT** | ↓ | ↑ | ↑ | ↑ | ↑ | ↓ | 0.048 | 15 | 37 | |
| 3 | **P01009** | alpha1-antitrypsin | **AAT** | ↓ | ↑ | ↑ | ↑ | ↑ | ↓ | 0.018 | 11 | 25 | |
| 4 | **P02671//P06870** | Fibrinogen alpha chain // Kallikrein1 | **FGA // KLK1** | ↑ | ↓ | ↓ | ↓ | ↓ | ↑ | 0.021 | 5 // 6 | 8 // 16 | |
| 5 | **P05452** | Tetranectin | **TNA** | ↑ | ↓ | ↓ | ↓ | ↓ | ↑ | 0.033 | 10 | 58 | |
| 6 | **P02760** | AMBP protein | **AMBP** | ↓ | ↓ | ↓ | ↓ | ↑ | ↑ | 0.012 | 12 | 29 | |
| 7 | **P13987** | CD59 glycoprotein | **CD59** | ↑ | ↓ | ↓ | ↓ | ↓ | ↑ | 0.0024 | 5 | 21 | |
| 8 | **P13987** | CD59 glycoprotein | **CD59** | ↑ | ↓ | ↓ | ↓ | ↓ | ↑ | 0.0022 | 5 | 21 | |
| 9 | **P13987** | CD59 glycoprotein | **CD59** | ↑ | ↓ | ↓ | ↓ | ↓ | ↑ | 0.03 | 4 | 21 | |
| 10 | **P13987** | CD59 glycoprotein | **CD59** | ↑ | ↓ | ↓ | ↓ | ↓ | ↑ | 0.0082 | 6 | 21 | |
| 11 | **P25311** | Zinc-alpha 2 glycoprotein | **AZGP1** | ↑ | ↑ | ↑ | ↑ | ↑ | ↓ | 0.016 | 8 | 22 | |
| 12 | **Q8WVN6 // P02766** | Secreted and transmembrane protein1 // Transthyretin | **SECTM1 // TTR** | ↓ | ↑ | ↑ | ↑ | ↑ | ↓ | 0.018 | 7 // 6 | 25 // 25 | |

**Table S4.** SRM-LC-MS/MS analysis conditions for those proteins confirmed in the validation phase with statistical signification (ANOVA <0.0001). Details of protein transitions (precursor and fragments masses), collision energy and peptide sequences are included.

| **Gene** | **Precursor peptide sequence** | **Precursor m/z** | **Fragmentor masses** | **Collision energy** | Fragmentor | Dwell time |
| --- | --- | --- | --- | --- | --- | --- |
| **CD59** | K.FEHCNFNDVTTR.L | 513.9 | 532.7 574.2 582.2 591.3 632.3 688.3 705.4 835.3 | 9.2 | 130 | 20 |
|  |  | 770.3 | 1064.4 1126.5 1163.5 | 23.7 | 130 | 20 |
| **AAT** | K.ITPNLAEFAFSLYR.Q | 548.0 | 552.8 685.4 734.4 903.5 | 10.5 | 130 | 20 |
| **TNA** | K.NWETEITAQPDGGK.T | 515.9 | 537.2 | 9.3 | 130 | 20 |
|  | MELWGAYLLLCLFSLLTQVTTEPPTQKPK.K | 1126.6 | 1207.7 1225.7 | 31.9 | 130 | 20 |

**Figure S1.** Representative image of 2D-DIGE gel.


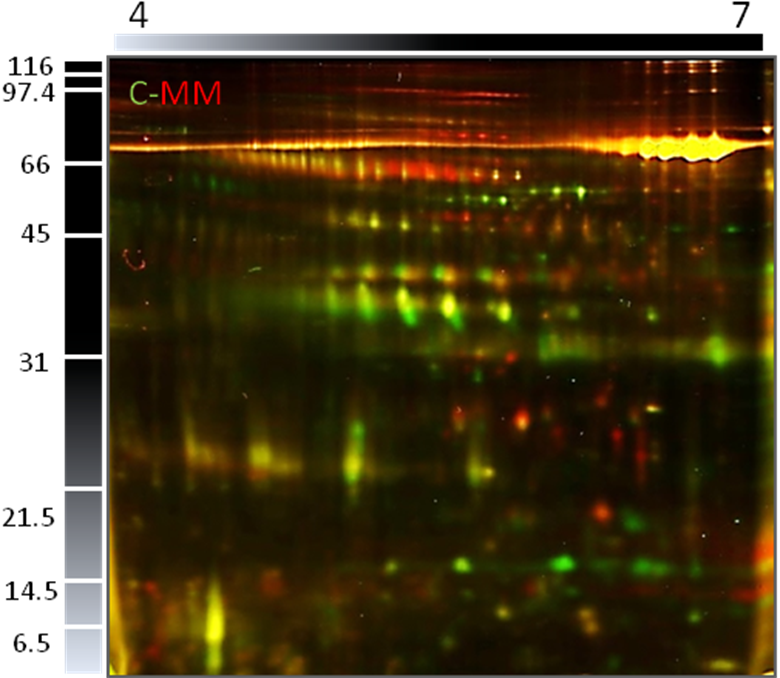


**Figure S2.** Principal component analysis (PCA) graph. Each dot represents a urine sample used in the discovery phase (DIGE analysis).


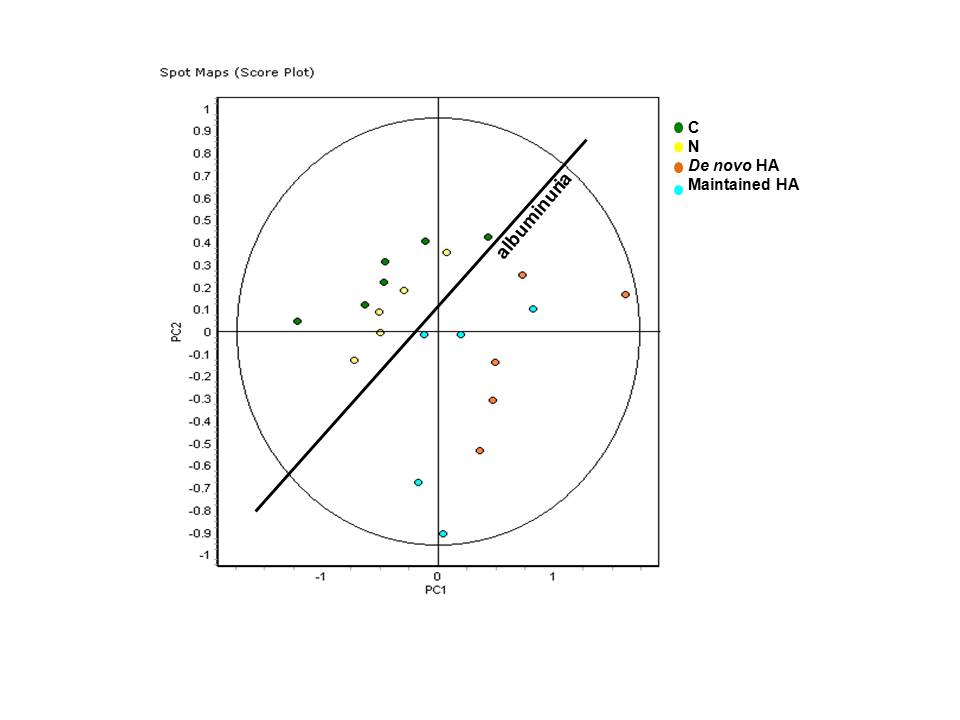

Supplement: Supplementary file 1 — 10.1186/s12933-016-0331-7 Representative image of 2D-DIGE gel. Figure S2. Principal component analysis (PCA) graph. Each dot represents a urine sample used in the discovery phase (DIGE analysis). Table S1. Baseline medication for those patients including in the discovery phase. Data are expressed as percentages (%). ACEi: angiotensin converting enzyme inhibitors; ARB: angiotensin receptor blockers. N: normoalbuminuria; dnHA: de novo high albuminuria; MHA: maintained high albuminuria. Table S2. Baseline medication for those patients including in the validation phase. Data are expressed as percentages (%). ACEi: angiotensin converting enzyme inhibitors; ARB: angiotensin receptor blockers. N: normoalbuminuria; dnHA: de novo high albuminuria; MHA: maintained high albuminuria. Table S3. Proteins identifiedper gel spot with significant alteration (one-way ANOVA). The table shows the number of unique peptides identified, % sequence coverage and trends observed for each protein between compared groups (increase or decrease in the group located in the upper part of the ratio). Two spots contain a mixture of two proteins each, thus changes in expression forthose spots cannot be attributed to any of the two proteins initially. When one protein was identified in several spots, observed variations between groups followed the same trend (e.g. CD59 and alpha-1-antitrypsin). Table S4. SRM-LCMS/MS analysis conditions for those proteins confirmed in the validation phase with statistical signification (ANOVA <0.0001). Details of protein transitions (precursor and fragments masses), collision energy and peptide sequences are included. [file 12933_2016_331_MOESM1_ESM.doc]
